# Combined transcranial and endoscopic endonasal approach for anterior skull base meningiomas: approach and reconstruction strategies

**DOI:** 10.3171/2025.10.FOCVID25157

**Published:** 2026-01-01

**Authors:** Stephen Whipple-Bones, Mark H. Tabor, Stephen T. Magill, Danielle Terrell, Andre Beer-Furlan

**Affiliations:** Departments of 1 Neurosurgical Oncology and; 2 Head and Neck Surgery, Moffitt Cancer Center, Tampa, Florida; and; 3 Department of Neurological Surgery, Northwestern University, Chicago, Illinois

**Keywords:** anterior skull base, meningioma, endoscopic, endonasal, reconstruction

## Abstract

Large anterior skull base meningiomas with sinonasal invasion pose a challenge not only for safe resection but also for tackling reconstruction of the anterior skull base. Here, in this video, the authors present the case of a 35-year-old female with a biopsy-proven WHO grade II meningioma who required a combined transcranial and endoscopic endonasal approach. This combined approach allowed for excellent tumor exposure, complete safe resection, and, importantly, durable and appropriate skull base reconstruction.

The video can be found here: https://stream.cadmore.media/r10.3171/2025.10.FOCVID25157

## Transcript

In this video, we will show a combined endoscopic transnasal and transcranial approach to a large anterior skull base and intranasal contiguous mass, discussing the best approach for this tumor through nuanced corridors and exposure-related reconstruction.

### 0:34 Clinical Presentation and Neuroimaging.

Our patient is a 35-year-old female who presented to our clinic with months of headaches, left eye pain, and double vision. She also had some loss of smell. She underwent neuroradiological investigation by an otolaryngologist and was found to have a large skull base/intranasal mass. They did an intranasal biopsy, and pathology confirmed CNS WHO grade II chordoid meningioma. The MRI showed a large paramedian intracranial component, more so on the left, as well as a significant contiguous intranasal component. Also note the small component that violates the medial wall of the left orbit, which is likely the source of her ocular symptoms. CT scan demonstrated the bony destruction of the frontal sinus, anterior skull base, and the medial wall of the left orbit.

### 1:34 Surgical Rationale.

The emphasis for this video is that a single approach to this tumor would not allow for a complete resection, safe complication avoidance, and appropriate reconstruction of the tumor-inflicted defects. Specifically, reconstruction is a key component to surgical planning, and specific local or regional tissue transfers must be considered before surgery.^1^ A transnasal route would allow access to the intranasal, orbital, and some of the intracranial components, but if smell preservation is key, then this approach would be less favored. Also, a traditional nasoseptal flap reconstruction might prove to be insufficient coverage for the skull base defects given the amount of tumor infiltrating the nose.^2^ Given this tumor invades through the posterior wall of the frontal sinus, the ability to reconstruct from below through an endonasal route would be exceedingly difficult, unsafe, and likely inadequate. A strictly transcranial approach would prove to allow good access to the anterior skull base, frontal sinus, and orbital components of the tumor, but downstream intranasal pathology would be left behind. Reconstruction with a pericranial graft would be sufficient for defect mitigation. The best tailored approach for this tumor, keeping in mind tumor location, resection goals, complication avoidance, and adequate reconstruction material and exposure, is combining transnasal and transcranial.^3,4^

### 2:50 Intraoperative Considerations.

When planning for the single-setting combined case, forethought must go into the positioning, exposure, and draping of the patient. In our case, the patient was positioned supine in three-point Mayfield fixation, and the bed turned 180° to accommodate both surgical teams. The head was held in slight extension and tilted to the right to allow for a better anterior skull base view and facilitate endonasal ergonomics. The bicoronal incision was planned and marked out. The cranial incision was prepped and draped in standard fashion, as well as the nose prepared for the endonasal portion. Split drapes were used to allow for better contouring of the two separate working areas. The endoscopic screens were positioned so both surgeons were able to view them easily, with the endonasal surgeon standing on the right side of the patient. It was decided beforehand that much of the cranial work would be done before beginning the endonasal portion, and then the joint work could be accomplished to finish the case.

### 3:47 Pericranial Harvest and Transcranial Resection.

A standard bicoronal incision was opened, and the superficial layers were elevated away from the pericranium. This dissection was carried all the way to the periorbita to allow for skull base bone cuts and better cosmetic reconstruction later. The pericranium was then elevated from the bone and protected with wet sponges for the remainder of the case. Bone cuts were made along the superior sagittal sinus in the midline and down at the keyhole bilaterally and carried along the roof of the orbit at the anterior skull base. Dura was opened along either side of the sinus. The tumor was then devascularized and debulked at the anterior skull base. Intraoperatively, the tumor was seen crossing the falx and infiltrating the cribriform plate on the right side as well, so smell preservation was unable to be achieved. The anterior skull base was drilled from above, and the dura cuts were taken down to the skull base, and the ethmoidal arteries are coagulated and divided, which is performed quicker from the transcranial route. Posterior wall of the frontal sinus and mucosa are removed from the bone flap/craniotomy. The remaining frontal sinus septations including supraorbital air cells are drilled down, creating a single cavity (transcranial Draf III). If any of the frontal sinus air cells are at risk of being blocked by the pericranial flap, then we remove the mucosa to avoid mucocele. If the air cell is communicating with the nasal side, then no efforts are made to remove mucosa.

### 5:07 Endoscopic Endonasal Resection and Reconstruction.

After accessing the nose, the tumor was encountered and electrocautery was used to begin debulking. The tumor was easily removed from the surrounding mucosa, and this was carried superiorly to the skull base. While most of the drilling was done from the transcranial corridor, the remainder of the skull base was drilled from the nose to connect the two corridors. As you can see, the cottonoid patties can be seen intracranially from the nasal endoscope. Our attention was then turned to the medial wall of the orbit, which demonstrated tumor infiltration radiographically. The tumor was resected away from the orbit from both transcranial and transnasal corridors until periorbita was seen and no tumor remained extraconally. Reconstruction consisted of layering the pericranial graft down from above to cover the skull base defect and continuing this as far posterior into the sphenoid sinus. The pericranial flap is rotated along the nasal side of the skull base and buttressed with a nasoseptal flap to rest up against the anterior skull base. The nasoseptal flap helps expedite mucosalization of the pericranial flap and holds it in place; thus, the pericranium is not sutured to any structures. The advantage of viewing this from the nose is you can assist in pulling the graft into the nose to allow for complete coverage of the defect. A dural substitute was also placed intracranially over the excised dura and sutured to the dural defect in a watertight fashion. Surgicel, dural sealant, and packing were then used in standard fashion to complete the reconstruction and repair.

### 6:35 Postoperative Imaging and Outcome.

The patient did well after surgery with resolution of her double vision. Postoperative imaging shows excellent resection of all components of the tumor; specifically, there is no residual seen in the orbit. The intracranial cavity did not demonstrate any diffusion restriction or residual contrast enhancement left behind. Bone window CT scan shows orbital decompression and removal of all the skull base bone affected by the tumor. The coronal again demonstrates the flush bone cuts at the skull base, which facilitate cosmetic reconstruction. The patient’s pathology came back again as CNS WHO grade II chordoid meningioma. Next-generation sequencing and methylation studies were performed, with the results seen here with no actionable mutations. The patient was offered adjuvant radiation treatments but opted for surveillance, given gross-total resection was achieved.

### 7:33 Summary of Surgical Goals.

The three major facets of surgical planning for this patient and her pathology were tumor resection, safety, and reconstruction. Following a patient-specific algorithm, the combined approach was best suited for this patient.^5^ The transcranial route provided access to the skull base and orbit with direct visualization for dissection and a route to harvest a pedicled pericranial graft for reconstruction. The addition of the transnasal corridor allowed complete, safe resection of the nasal components of the tumor, a bottom-up view of the skull base and orbit to assure complete resection, and finally it ensured excellent skull base reconstruction.

### 8:10 Other Examples.

Here we show two other examples of large anterior skull base meningiomas that were tackled similarly with combined approaches. The CT scans also demonstrate the extent of the anterior skull base bony removal, including the cribriform plate of the ethmoid, the frontal bone surrounding the cribriform plate, and the frontal sinus obliteration. Again, given the specific patient’s symptoms, tumor locations, and surgical goals including durable reconstructions, combined approaches were also used with great results.

## Disclosures

Dr. Tabor reported consulting fees from Medtronic and speaker fees from Sanofi outside the submitted work. Dr. Magill reported personal fees from Stryker and Black Forest Medical Group outside the submitted work.

## Author Contributions

Primary surgeon: Beer-Furlan, Tabor. Assistant surgeon: Terrell. Editing and drafting the video and abstract: Beer-Furlan, Whipple-Bones, Tabor. Critically revising the work: Beer-Furlan, Whipple-Bones, Tabor, Magill. Reviewed submitted version of the work: Beer-Furlan, Whipple-Bones, Tabor, Magill. Approved the final version of the work on behalf of all authors: Beer-Furlan. Supervision: Beer-Furlan.

## Supplemental Information

### Patient Informed Consent

The necessary patient informed consent was obtained in this study.
